# Differentiation of Lung Cancer, Empyema, and Abscess Through the Investigation of a Dry Cough

**DOI:** 10.7759/cureus.896

**Published:** 2016-11-24

**Authors:** Brittany Urso, Scott Michaels

**Affiliations:** 1 College of Medicine, University of Central Florida; 2 FM Medical, Inc.

**Keywords:** lung abscess, empyema, lung infection, pneumonia, thoracotomy, lobectomy, pulmonology, respiratory infections

## Abstract

An acute dry cough results commonly from bronchitis or pneumonia. When a patient presents with signs of infection, respiratory crackles, and a positive chest radiograph, the diagnosis of pneumonia is more common. Antibiotic failure in a patient being treated for community-acquired pneumonia requires further investigation through chest computed tomography. If a lung mass is found on chest computed tomography, lung empyema, abscess, and cancer need to be included on the differential and managed aggressively.

This report describes a 55-year-old Caucasian male, with a history of obesity, recovered alcoholism, hypercholesterolemia, and hypertension, presenting with an acute dry cough in the primary care setting. The patient developed signs of infection and was found to have a lung mass on chest computed tomography. Treatment with piperacillin-tazobactam and chest tube placement did not resolve the mass, so treatment with thoracotomy and lobectomy was required. It was determined through surgical investigation that the patient, despite having no risk factors, developed a lung abscess.

Lung abscesses rarely form in healthy middle-aged individuals making it an unlikely cause of the patient's presenting symptom, dry cough. The patient cleared his infection with proper management and only suffered minor complications of mild pneumoperitoneum and pneumothorax during his hospitalization.

## Introduction

Determining the etiology of an acute dry cough can be an easy diagnosis such as bronchitis or pneumonia; however, it can also develop from other etiologies. Initial workup is often not needed; however, if there are other findings on physical exam, such as lung crackles, fever, tachycardia, or shortness of breath which may indicate pneumonia, then a chest radiograph (CXR) is indicated [1]. If symptoms persist and worsen, antibiotics are necessary. The first line treatment for community-acquired pneumonia requires coverage of *Streptococcus pneumoniae* strains with an oral regimen of azithromycin, clarithromycin, or doxycycline [1-2]. If the treatment fails, a respiratory fluoroquinolone such as levofloxacin should be considered [1-2]. After subsequent treatment failure, then chest computed tomography (CT) should be used to further evaluate the etiology of the lung disease [1]. Bronchoscopy with bronchoalveolar lavage (BAL) is also helpful in investigating underlying etiologies within the bronchi [3]. Etiologies, such as infection and malignancy, should be considered and a complete blood count with complete metabolic panel should be ordered.

If a lower lung mass is identified on the chest CT following symptoms of a lung infection, a lung empyema should be high on the differential. Lung empyemas occur as a complication of pneumonia in one to two percent of all community-acquired pneumonia [4]. Lung tumor and abscess should also be considered due to similar presentations. An empyema is a collection of pus within the pleural space that occurs secondary to pneumonia. Pneumonia increases the interstitial fluid within the lung and if the fluid is non-sterile, an immune response is triggered causing loculation and fibrin deposition [5]. As a result, on CXR, an empyema should stack in the same distribution as a parapneumonic effusion [6]. A lung abscess occurs most commonly due to aspiration pneumonia; however, it can also occur secondary to bacteremia, direct inoculation of infected tissue, or acute necrotizing pneumonia [3]. Patients at risk for lung abscesses are patients who are alcoholics or drug abusers, have a seizure disorder, have undergone recent general anesthesia, or have a nasogastric or endotracheal tube [7]. Most abscesses resulting from aspiration pneumonia contain oral flora such as *Prevotella, Peptostreptococcus, Fusobacterium*, or *Bacteroides* [7]. If the abscess results from other causes, then *S. aureus, H. influenzae, *and *S. pneumoniae* are likely pathogens [7]. As in an empyema, abscesses occur when the body’s immune system tries to wall off the infection [3].

Once an empyema or abscess has developed, the physician must decide if it is safe for the patient to let the mass resolve independently [3, 8]. If the patient’s symptoms are not severe, a four to six week trial of antibiotics can be given [3]. There is no empiric antibiotic regimen for the treatment of thoracic empyema or abscess. If the patient fails treatment or is experiencing symptoms of fever, tachycardia, hypotension, or tachypnea, then pleural drainage is required [3]. The least aggressive method of pleural drainage is CT-guided tube thoracostomy with small bore catheter placement [8-10]. Any fluid drained from the mass should be cultured and assessed for pH, lactate dehydrogenase, glucose level, and cell count [10]. If the chest tube is placed and the mass persists, then another method of pleural drainage must be used [8, 10]. Masses that are solid or loculated often resist drainage [8, 10]. Additionally, empyemas and abscesses may reseal off following chest tube placement due to deposition of fibrin [9].

Open thoracotomy is a more invasive method of lung mass drainage. This allows the surgeon to view the mass and determine other possible diagnoses. If the mass is an empyema or abscess, the surgeon would create an open incision in the lower border of the mass and place a chest tube [10]. If the mass is not an empyema or abscess and another pathology is identified, then the surgeon can complete a lobectomy or resection, as needed [8, 10].

Overall, an acute cough may lead to a simple diagnosis or it can lead to a battery of tests and a large workup. The goal of this case study is to show the investigation and management of an acute dry cough.

## Case presentation

**Figure 1:**
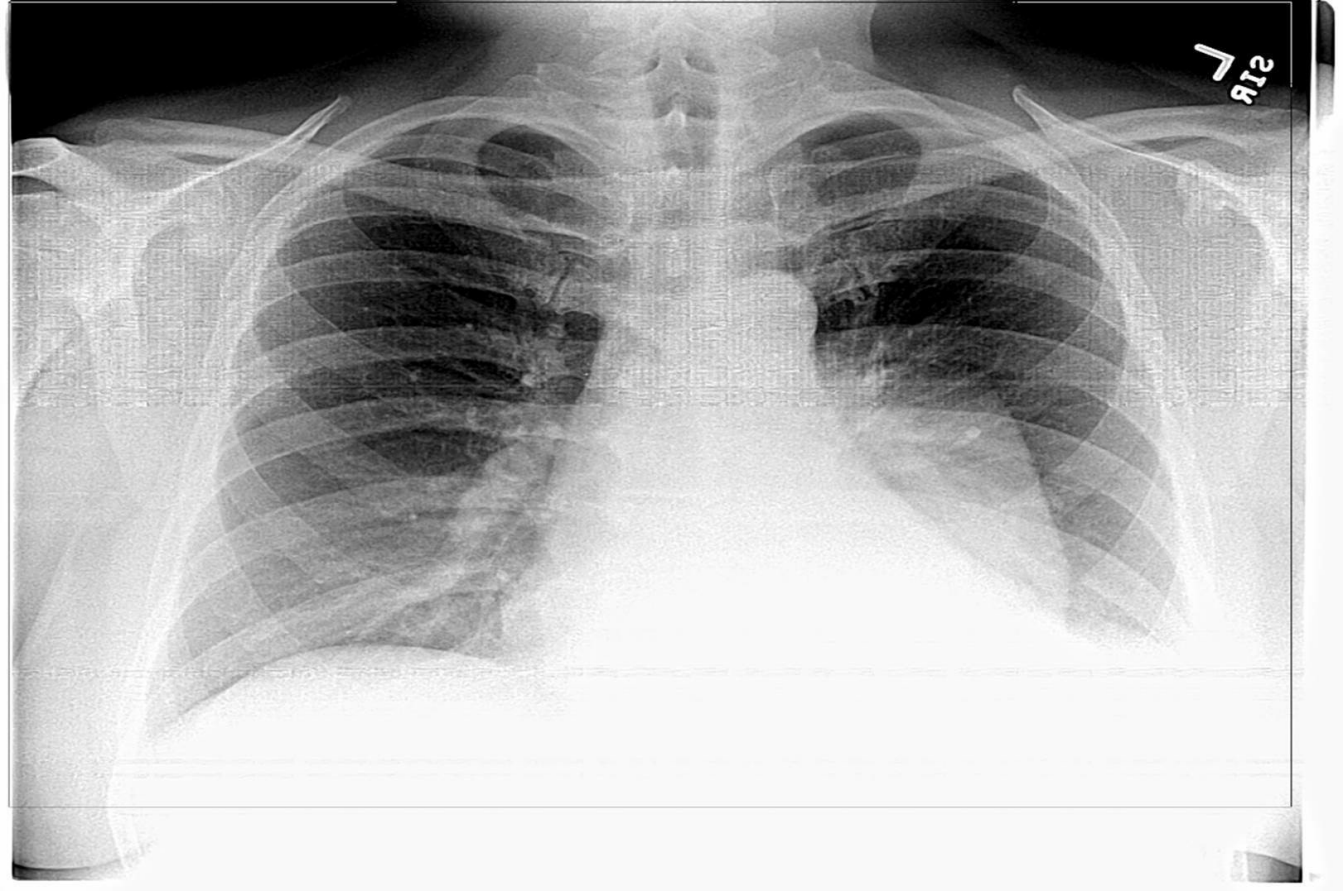
Chest radiograph with left-sided mass Chest radiograph in PA view showing a left-sided mass and pleural effusion. The mass is well-marginated and rounded without a meniscus.

A 55-year-old Caucasian male with a history of obesity, hypercholesterolemia, recovered alcoholism, and hypertension presented to his primary care physician with complaints of fatigue and a dry, non-productive cough. The patient never used tobacco, but did drink ten beers per day prior to quitting in April 2016. He worked in sales and denied any exposure to fumes, gasses, or smoke. On physical exam, there were no significant findings, so the patient was told to follow-up with the physician if his condition worsened. On follow-up, bibasilar crackles were heard in lower lung fields and the patient complained of worsening fatigue. A CXR was ordered, and the patient was put on a four-day course of azithromycin-amoxicillin with clavulanic acid (Figure 1).

The patient failed the first round of antibiotics and was given a methylprednisolone injection, as well as a five-day course of 750 mg levofloxacin due to new onset low-grade fever and worsening fatigue. The patient was also sent for an outpatient chest CT scan that day and was told to follow-up the next day if he was not feeling better. The next day, the patient was admitted to the hospital with fever, severe fatigue, and dry cough. His chest CT revealed an 11.3 x 7.7 x 6.6 cm mass, which was pleural, contiguous, and appeared to be compressing the left lung parenchyma. The mass did not seem to contain any calcifications or foreign bodies. Pulmonology and cardiothoracic surgery were consulted.

The patient underwent diagnostic bronchoscopy with bronchoalveolar lavage to investigate intrabronchial causes of infection and abscess. Bronchoscopy revealed slight pus in the left lower bronchi; however, it was not significant. The leading diagnosis at the time was lung empyema with parapneumonic effusion so the patient was managed with empiric treatment of piperacillin-tazobactam for broad coverage. The patient was feeling significantly better on this treatment; however, the empyema was not responding. As a result, the patient was sent to interventional radiology for CT-guided percutaneous drain placement. An 8 French catheter was placed in the left pleural space draining 200 mL of green, purulent fluid. The chest tube was connected to a pleural vac under low wall suction. Eight milliliters of pus were sent for culture and microscopic analysis. The specimen revealed numerous white blood cells and a few gram-negative bacilli. At 24 hours, the specimen was not showing growth on bacterial or fungal culture. The chest tube was left in for five days. It initially drained 124 mL of fluid, but then consistently drained less than 50 mL per day. On re-evaluation of the empyema by CXR, it was determined that the left thorax opacification was unchanged.

At this time, cardiothoracic surgery and pulmonology decided that thoracotomy would be the best option to remove the lower left thorax mass. Additionally, cardiothoracic surgery stated that the mass might be an infected bronchogenic cyst, tumor, or lung abscess. The cardiothoracic surgeon stated that the mass was most likely not an empyema due to the rounded shaped of the mass, since a lung empyema usually follows the shape of the pleura.

The thoracotomy occurred on day seven of hospitalization. The cardiothoracic surgeon noted significant inflammatory adhesions between the lung pleura and the chest wall, as well as between the left lower lung lobe and the aorta, which had to be dissected away carefully. A left lower lobectomy was completed, and lymph nodes as well as a solid mass in the upper left lobe were excised. All tissue samples were sent to pathology. The patient tolerated the surgery well and left the operating room with two 28 Argyle chest tubes attached to suction. Pathology determined that the lung mass was an abscess of unknown etiology and that there was no evidence of cancer.

Postoperatively, the patient was stable and was managed on morphine for pain. By postoperative day three, the patient had not had a bowel movement and on physical exam, his abdomen was hard and distended with absent bowel sounds. The patient developed ileus but was experiencing no nausea or vomiting. The patient was continued on a bowel prep; however, the next day he began vomiting. As a result, a nasogastric tube was placed for bowel decompression and metoclopramide was given to encourage gut motility.

Additionally, on CXR, slight pneumothorax and pneumoperitoneum were noted. The patient was monitored for increasing leukocytosis, fever, and respiratory distress; however, these finding resolved. The postoperative ileus resolved with treatment and the patient was transitioned onto oral metronidazole and cefdinir prior to being discharged. The patient was discharged from the hospital after eighteen days of inpatient treatment. Informed consent was obtained from the patient for this study.

## Discussion

Every day patients present to their primary care physicians with the complaint of an acute cough. Most of the time, the cough is the result of a new medication regimen, asthma, or a recent cold. It is rare for these symptoms to indicate severe disease in a normally healthy middle-aged adult. Despite this, it is imperative that physicians keep an open differential diagnosis because an acute cough can indicate lung cancer or lung abscess.

Our patient was fairly healthy and lacked any risk factors for a lung abscess. Due to his previous alcohol usage, aspiration pneumonia was considered; however, it was believed to be unlikely due to the patient quitting drinking in April 2016. Also, the patient denied having any alcoholic blackouts. At initial presentation, lung abscess was at the very bottom of the differential with pneumonia more likely. Even after chest CT and bronchoscopy, a lung empyema was believed to be significantly more likely than a lung abscess, despite the more rounded shape of the lung mass. Due to the similar management of both lung empyema and lung abscess, consisting of a broad antibiotic spectrum, thoracostomy, and aggressive thoracotomy following treatment failure, the patient was managed well and his infection was eradicated.

Patients experiencing persistent symptoms of a dry cough and infection need to be managed aggressively to avoid severe sepsis or septic shock. Primary care physicians need to be able to recognize when a patient can be managed on an outpatient basis and when they need to admit the patient and receive a consult from specialists. Though a dry cough often has benign etiology, it is imperative that even the unlikely causes of a cough, such as lung abscess, are not excluded.

## Conclusions

The differentiation of lung abscess, empyema, and cancer are not always clear when evaluating a patient. Pneumonia can usually be ruled in or out from the findings of a chest radiograph, CT, or bronchoscopy, but determining whether or not a mass is cancer or infection requires more studies. The absence of air-fluid levels on chest CT or CXR further complicates diagnosis, requiring the patient to undergo pleural drainage if the patient's condition is not improving. Additionally, diagnostic bronchoscopy can be used to determine whether or not the patient's symptoms stem from an intrabronchial process such as pneumonia.

Broad antibiotic coverage with antibiotics such as piperacillin-tazobactam is needed in patients who are believed to have an empyema or lung abscess. Thoracostomy with suction may be enough for mass drainage, but in situations where it is not, a thoracotomy can be considered. Patients typically undergo thoracotomy if their symptoms are severe enough that they cannot wait for the mass to resolve with a prolonged course of antibiotics.

Thoracotomy is a very invasive procedure, but it is extremely useful in resolving the underlying infection. Our patient had a lung abscess that destroyed the left lower lobe of the lung. The necrotized lung tissue acted as a nidus for infection, which promoted lung abscess development. Without aggressive treatment, the patient could have developed life-threatening complications such as pneumonia or sepsis. In conclusion, the diagnosis of lung abscess, lung empyema, and cancer are not always clear. When a patient is experiencing signs and symptoms of severe infection, the health care team must act aggressively to determine the underlying etiology.

## References

[1] Mandell LA, Wunderink RG, Anzueto A, Bartlett JG, Campbell GD, Dean NC, Dowell SF, File TM, Musher DM, Niederman MS, Torres A, Whitney CG (2007). Infectious Diseases Society of America/American Thoracic Society consensus guidelines on the management of community-acquired pneumonia in adults. Clin Infect Dis.

[2] Bernstein JM (1999). Treatment of community-acquired pneumonia--IDSA guidelines. Infectious Diseases Society of America. Chest.

[3] Kuhajda I, Zarogoulidis K, Tsirgogianni K, Tsavlis D, Kioumis I, Kosmidis C, Tsakiridis K, Mpakas A, Zarogoulidis P, Zissimopoulos A, Baloukas D, Kuhajda D (2015). Lung abscess-etiology, diagnostic and treatment options. Ann Transl Med.

[4] Schattner A, Dubin I, Gelber M (2016). Double jeopardy – concurrent lung abscess and pleural empyema. QJM.

[5] Strange C, Tomlinson JR, Wilson C, Harley R, Miller KS, Sahn SA (1989). The histology of experimental pleural injury with tetracycline, empyema, and carrageenan. Exp Mol Pathol.

[6] Kearney SE, Davies CW, Davies RJ, Gleeson FV (2000). Computed tomography and ultrasound in parapneumonic effusions and empyema. Clin Radiol.

[7] Kwong JC, Howden BP, Charles PG (2011). New aspirations: the debate on aspiration pneumonia treatment guidelines. Med J Aust.

[8] Muhammad MI (2012). Management of complicated parapneumonic effusion and empyema using different treatment modalities. Asian Cardiovasc Thorac Ann.

[9] Colice GL, Curtis A, Deslauriers J, Heffner J, Light R, Littenberg B, Sahn S, Weinstein RA, Yusen RD (2000). Medical and surgical treatment of parapneumonic effusions: an evidence-based guideline. Chest.

[10] Scarci M, Abah U, Solli P, Page A, Waller D, van Schil P, Melfi F, Schmid RA, Athanassiadi K, Sousa Uva M, Cardillo G (2015). EACTS expert consensus statement for surgical management of pleural empyema. Eur J Cardiothorac Surg.

